# Generation and Comprehensive Analysis of Host Cell Interactome of the PA Protein of the Highly Pathogenic H5N1 Avian Influenza Virus in Mammalian Cells

**DOI:** 10.3389/fmicb.2017.00739

**Published:** 2017-04-28

**Authors:** Zhao Gao, Jiao Hu, Yanyan Liang, Qian Yang, Kun Yan, Dong Liu, Xiaoquan Wang, Min Gu, Xiaowen Liu, Shunlin Hu, Zenglei Hu, Huimou Liu, Wenbo Liu, Sujuan Chen, Daxin Peng, Xin-an Jiao, Xiufan Liu

**Affiliations:** ^1^Animal Infectious Disease Laboratory, School of Veterinary Medicine, Yangzhou UniversityYangzhou, China; ^2^Jiangsu Co-innovation Center for Prevention and Control of Important Animal Infectious Diseases and Zoonosis, Jiangsu Key Laboratory of Zoonosis, Yangzhou UniversityYangzhou, China; ^3^Jiangsu Key Laboratory of Zoonosis, Jiangsu Co-Innovation Center for Prevention and Control of Important Animal Infectious Diseases and Zoonoses, Yangzhou UniversityYangzhou, China

**Keywords:** H5N1 IAV, PA protein, virus-host interaction, pathogenesis, mammalian adaptation

## Abstract

Accumulating data have identified the important roles of PA protein in replication and pathogenicity of influenza A virus (IAV). Identification of host factors that interact with the PA protein may accelerate our understanding of IAV pathogenesis. In this study, using immunoprecipitation assay combined with liquid chromatography-tandem mass spectrometry, we identified 278 human cellular proteins that might interact with PA of H5N1 IAV. Gene Ontology annotation revealed that the identified proteins are highly associated with viral translation and replication. Further KEGG pathway analysis of the interactome profile highlighted cellular pathways associated with translation, infectious disease, and signal transduction. In addition, Diseases and Functions analysis suggested that these cellular proteins are highly related with Organismal Injury and Abnormalities and Cell Death and Survival. Moreover, two cellular proteins (nucleolin and eukaryotic translation elongation factor 1-alpha 1) identified both in this study and others were further validated to interact with PA using co-immunoprecipitation and co-localization assays. Therefore, this study presented the interactome data of H5N1 IAV PA protein in human cells which may provide novel cellular target proteins for elucidating the potential molecular functions of PA in regulating the lifecycle of IAV in human cells.

## Introduction

Influenza A virus (IAV) is one of the most important pathogens that causes acute respiratory disease and is responsible for relatively high morbidity and mortality every winter. In addition, IAV is a very adaptable virus and can infect a wide range of hosts (Webster et al., 1992; Olsen et al., 2006). IAV has an enveloped negative-strand RNA genome that is composed of eight viral RNA segments. IAV genome encodes at least 17 viral proteins, including 10 initially identified proteins (PB2, PB1, PA, HA, NP, NA, M1, M2, NS1, and NS2) and seven newly identified proteins PB1-F2 (Chen et al., 2001), PB1-N40 (Wise et al., 2009), PA-X (Jagger et al., 2012), PA-N155, PA-N182 (Muramoto et al., 2013), M42 (Wise et al., 2012), and NS3 (Selman et al., 2012). The PA protein is the third subunit of the trimeric polymerase complex which has multiple functions in the life cycle and pathogenesis of IAV. The N-terminal of PA possesses the endonuclease activity, cap binding, and promoter binding functions that impact the viral transcription and replication (Hara et al., 2006). Accumulating studies have also shown that adaptive mutations in PA are essential for IAV to cross host barrier (Seyer et al., 2012; Sun et al., 2014; Song et al., 2015). In addition, the PA protein is also a key player in virulence-determining of IAV (Hulse-Post et al., 2007; Song et al., 2011; Hu et al., 2013a) as well as host protein shut off (Desmet et al., 2013; Hu et al., 2013a; Llompart et al., 2014). However, the potential mechanism associated with the roles of PA in the life cycle and pathogenesis of IAV is largely unknown.

Numerous studies have demonstrated that IAV heavily relies on host cellular proteins to complete its life cycle and pathogenicity (Hale et al., 2006; Gack et al., 2009; Cho et al., 2013; Gao et al., 2015). The protein-protein interaction is a crucial manner to maintain the connection between host and IAV. Several studies have identified some host factors interacting with the influenza PA protein. HAX1, a cytoplasmic protein with anti-apoptotic function, is associated with the nuclear localization signal domain of PA (Hsu et al., 2013). In IAV-infected cells, HAX1 can impede nuclear accumulation of PA and inhibit IAV replication, indicating that host cells use HAX1-PA as a defense mechanism to limit IAV infection. In addition, hCLE is another interacting partner of PA through binding with two domains of PA (amino acid residue 493–512 and 557–574), and this interaction involves in modulating the RNA polymerase II activity (Huarte et al., 2001; Pérez-González et al., 2006). In the process of influenza virus infection, the hCLE-PA interaction contributes to the increment of viral polymerase activity, viral RNA transcription and replication, virus titer, and viral particle production (Rodriguez et al., 2011). PA also has a close interaction with MCM complex that is considered to be a host regulator of viral genome replication (Kawaguchi and Nagata, 2007). Furthermore, other host proteins, such as RanBP5, AIFMI, NPM, and RIG-I also have been identified as interacting partners of PA and influence the viral life cycle of IAV (Deng et al., 2006; Mayer et al., 2007; Bradel-Tretheway et al., 2011; Li et al., 2014). However, based on the important roles of PA in the life cycle of IAV and close interplay between PA and host machinery, other potential host factors interacting with the PA protein still need to be identified.

In this study, immunoprecipitation (IP) technique coupled with liquid chromatography-tandem mass spectrometry (LC-MS/MS) was used to characterize the host cellular proteins interacting with the PA protein of H5N1 IAV. We identified two hundred and seventy eight human cellular proteins as interacting factors with PA. Further in-depth functional analysis suggested that most of these proteins involve in viral replication, gene expression and viral infectious cycle. Moreover, Kyoto Encyclopedia of Genes and Genomes (KEGG) pathway analysis also demonstrated the crucial roles of these proteins in translation, infectious disease, and signal transduction. Of note, we selected two host proteins, nucleolin (NCL), and eukaryotic translation elongation factor 1-alpha 1 (eEF1A1) and confirmed their interaction with the PA protein using co-IP and co-localization tests. However, further studies are still needed to explore the potential roles of these two proteins in the life cycle of IAV.

## Materials and methods

### Ethics statement

All experiments involving live viruses were performed in negative-pressure isolators with HEPA filters in a biosafety level 3 (BSL3) facilities in accordance with the institutional biosafety manual.

### Viruses and cells

Highly pathogenic H5N1 strain A/Chicken/Jiangsu/k0402/2010(CK10) that shows high virulence in mouse was isolated from a dead chicken (Hu et al., 2013b). A549 (human type II alveolar epithelial), 293T (human embryonic kidney) and MDCK (Madin-Darby canine kidney) cells were maintained in Dulbecco's modified Eagle's medium (DMEM; Life Technologies) supplemented with 10% fetal calf serum (FCS; Life Technologies) and 50 KU/L antibiotics and were cultured at 37°C under 5% CO_2_.

### Construction of plasmids

The PA gene was amplified using high fidelity DNA polymerase (Invitrogen) based on CK10 cDNA and cloned into pCDNA3.1 vector to generate pCDNA-PA. The NCL and eEF1A1 genes were amplified from A549 cells. The sequences of NCL and eEF1A1 have aligned with sequences from GenBank (accession No: NM005381.2, KJ891086). The NCL and eEF1A1genes were cloned into the pires-hrGFP-1a vector (with a 3 × Flag tag in the C-terminus) to generate pires-hrGFP-1a-NCL and pires-hrGFP-1a-eEF1A1, respectively. Meanwhile, these two genes were also cloned into the pCDNA3.1-Myc-C vector (with a 3 × Myc tag in the C-terminus) to generate pCDNA-Myc-NCL and pCDNA-Myc-eEF1A1, respectively. All the positive clones were further validated by sequencing.

### Western blot analysis

Protein samples were separated by electrophoresis on 10% (w/v) SDS-PAGE and transferred to polyvinylidene fluoride (PVDF) membranes (Bio-Rad). The membranes were then blotted with corresponding antibodies. Subsequently, the membranes were washed four times with TBST (0.05% tween-20 in Tris-buffered Saline) and incubated with horseradish peroxidase (HRP) conjugated goat-anti-mouse (Sigma-Aldrich) or goat-anti-rabbit IgG (Sigma-Aldrich). The enhanced chemiluminescence (ECL) system (Thermo) was utilized to detect the blotted proteins.

### Determination of the PA expression level

A549 cells were seeded in 6-well plates and cultured overnight. Then the cells were infected with CK10 virus at a multiplicity of infection (MOI) of 1. CK10-infected A549 cells were harvested at 12, 24, 36, 48, and 60 h post-infection (p.i.). The protein samples were subjected to western blot assay using anti-PA antibody and anti-β-actin antibody subsequently. The protein bands were analyzed by Image J.

### Statistical analysis

Statistical analysis was performed using the SPSS statistics software. The independent samples *T*-test was used for data analysis. *p* < 0.05 was considered as significant.

### IP and co-IP

For IP, CK10-infected A549 cells were lysed in IP buffer and incubated at 4°C on a shaker for 30 min, followed by centrifugation at 12,000 rpm for 20 min. A total of 600 μl of the supernatant was incubated with anti-PA ployclonal antibody (GeneTex) or the irrespective IgG (Beyotime) at 4°C overnight. Protein A+G Agarose beads (Beyotime) were then added, and the mixture was incubated with gentle rocking at 4°C for 5 h. The beads were washed five times with cold IP buffer and eluted with Glycline-HCL (PH = 3.0) for further LC-MS/MS analysis.

For co-IP, the 293T cells were co-transfected with pCDNA-PA or pCDNA3.1 alone and pires-hrGFP-1a-NCL, pires-hrGFP-1a-eEF1A1, or pires-hrGFP-1a, respectively. The transfected cells were then lysed in IP buffer at 48 h post-transfection. The cells lysates were precipitated with appropriate antibodies in conjunction with beads as described above. The beads were then washed five times with cold IP buffer and boiled with 5 × SDS loading buffer (Cwbio) for 5 min. The immunoprecipitated proteins were then detected by western blot.

### LC-MS/MS analysis and protein identification

The LC-MS/MS was performed on a mass spectrometer coupled to Easy nLC (Thermo Fisher Scientific). A volume of 6 μl of each fraction was injected for nanoLC-MS/MS analysis. The peptide mixture was loaded onto a C18-reversed phase column (Thermo Scientific Easy Column, 2 cm long, 100 μm inner diameter,5 μm resin) in buffer A (0.1% Formic acid) and separated with a linear gradient of buffer B (80% acetonitrile and 0.1% Formic acid) at a flow rate of 300 nL/min controlled by IntelliFlow technology over 140 min. MS data was acquired using a data-dependent on Top10 method dynamically choosing the most abundant precursor ions from the survey scan (300–1800 m/z) for HCD fragmentation. Determination of the target value is based on predictive Automatic Gain Control (pAGC). Dynamic exclusion duration was 60 s. Survey scans were acquired at a resolution of 70,000 at m/z 200 and resolution for HCD spectra was set to 17,500 at m/z 200. Normalized collision energy was 30 eV and the underfill ratio, which specifies the minimum percentage of the target value likely to be reached at maximum fill time, was defined as 0.1%. The instrument was run with peptide recognition mode enabled. MS/MS spectra were searched using MASCOT engine (Matrix Science, version 2.2) against Universal Protein (UniProt) database.

### Bioinformatics analysis

The functional annotation and classification of all the identified proteins were performed using Gene Ontology (GO) analysis tool in the Database for Annotation Visualization and Integrated Discovery (DAVID) (version 6.7). KEGG pathway database was used for pathway analysis. The Protein-Protein interaction network was constructed using the Cytoscape software. The Diseases and Functions analysis was conducted using Ingenuity Pathway Analysis (IPA) software.

### Confocal microscopy analysis

293T cells were seeded on coverslips in 24-well plates and cultured overnight, then co-transfected with pCDNA-PA or the empty vector (pCDNA3.1) alone and pCDNA-Myc-NCL, pCDNA-Myc-eEF1A1, or the empty vector (pCDNA-Myc-C), respectively. At 24 h post-transfection, the cells were fixed with 4% paraformaldehyde for 30 min at room temperature, and permeabilized with 0.1% Triton-X-100 for 15 min. After blocking in 5% bovine serum albumin (BSA) for 30 min, the cells were incubated for 1 h with the primary antibodies at 37°C. After washing in PBS for three times, the cells were stained with Alexa Fluor 555-conjugated goat anti-rabbit (Beyotime) and FITC-conjugated goat anti-mouse secondary antibodies (Beyotime) for 1 h at 37°C. After washing in PBS for three times, cell nuclei were stained with DAPI (Beyotime). The cells were then observed by using the Leica TCS SP8 STED 3X confocal microscope.

## Results

### Determination of the peak of PA expression in the process of IAV infection

**Figure 1:**
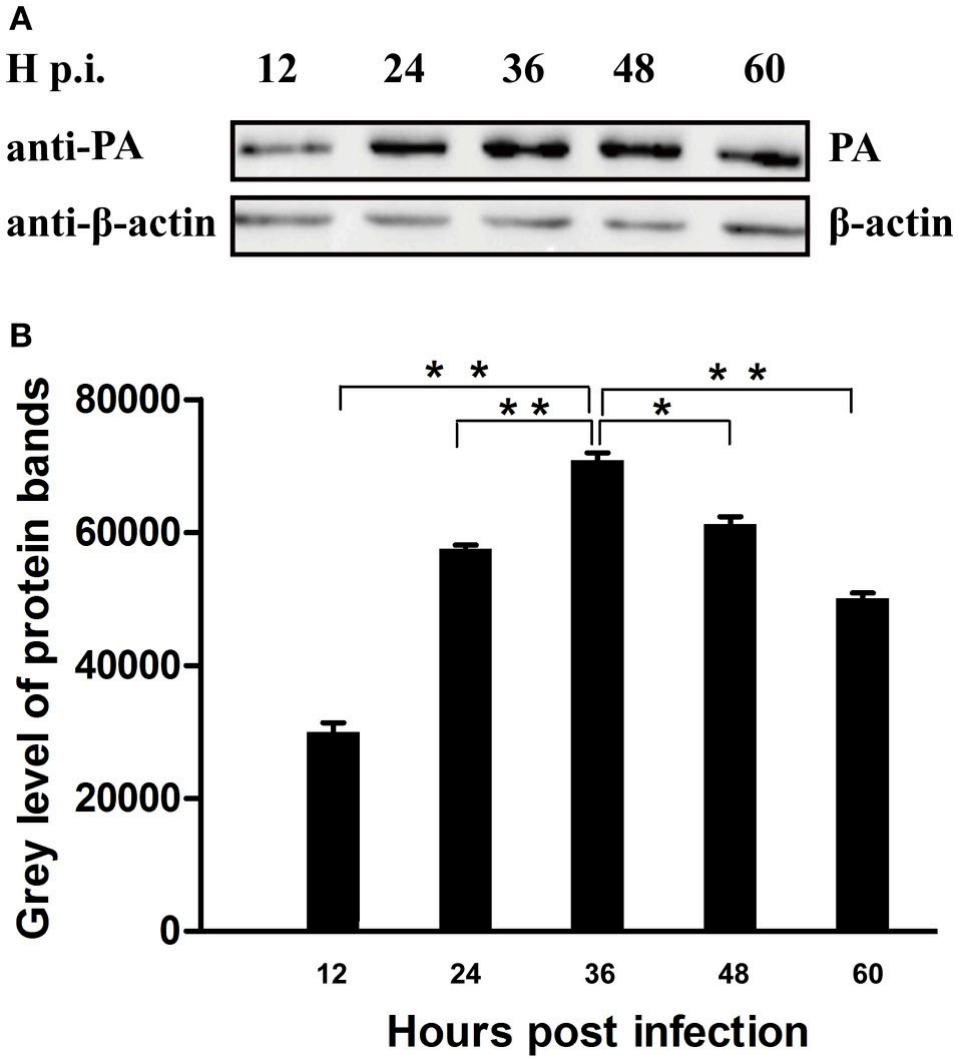
**Determination of the peak of PA expression in the process of IAV infection. (A)** Cell lysate from CK10-infected A549 cells at indicated time points were subjected to Western blot using anti-PA antibody and anti-β-actin antibody. Three independent experiments were perfomed and this is the representative data. **(B)** The Image J software was used to determine the expression level of the PA protein. Values shown are the gray level of the PA protein bands of western blot experiments ± SD of the results from three independent experiments. ^*^p < 0.05 and ^**^p < 0.01, when compared to the indicated group.

To determine the peak of PA expression, A549 cells that infected with CK10 virus at a MOI of 1 were harvested at 12, 24, 36, 48, and 60 h p.i. and the samples were then subjected to western blot assay by using anti-PA antibody as first antibody. At the same time, the expression patttern of β-actin protein was served as control. Three independent experiments were performed. The western blot protein bands were analyzed by Image J subsequently. And the results showed that the expression level of PA increased gradually in the process of CK10 infection, and reached a peak at 36 h p.i. The expression level of PA at 36 p.i. was significantly higher than those of other time points (Figures 1A,B). Therefore, we then collected the samples at 36 h p.i. for subsequent interactome analysis.

### Identification of host cellular proteins interacting with the PA protein

To efficiently precipitate the PA protein from the virus-infected cells and identify the host proteins that interact with PA, A549 cells were infected with CK10 at a MOI of 1. The cells were then harvested at 36 h p.i. and were immunopricicipitated with ployclonal antibody against PA or the irrespective antibody as control. Three independent experiments were carried out for both experimental group and control group. Subsequently, all samples were subjected to LC-MS/MS analysis. In addition, all proteins present in the negative controls were excluded and only proteins appeared at least twice in the triplicate analyses were reserved to generate interaction data. As a result, 278 proteins specifically precipitated with PA antibody were detected by LC-MS/MS analysis when compared to those precipitated with the irrespective IgG. A detailed summary of these proteins were given in Table 1 and Table S1.

**Table 1:** **PA-host interacting proteins in human cells (selected proteins)**.

**GenBank accession**	**Gene symbol**	**Protein name**
P68104	eEF1A1	Elongation factor 1-alpha 1
P19338	NCL	Nucleolin
P08238	HSP90AB1	Heat shock protein HSP 90-beta
Q00839	HNRNPU	Heterogeneous nuclear ribonucleoprotein U
P19474	TRIM21	E3 ubiquitin-protein ligase TRIM21
P12814	ACTN1	Alpha-actinin-1
P60842	eIF4A1	Eukaryotic initiation factor 4A-I
H0Y3T0	ATRX	Transcriptional regulator ATRX
P23528	CFL1	Cofilin-1
Q9BVA1	TUBB2B	Tubulin beta-2B chain
P10914	IRF1	Interferon regulatory factor 1
P0C869	PLA2G4B	Cytosolic phospholipase A2 beta
Q99665	IL12RB2	Interleukin-12 receptor subunit beta-2
Q13162	PRDX4	Peroxiredoxin-4
P18124	RPL7	60S ribosomal protein L7
P25705	ATP5A1	ATP synthase subunit alpha
Q96J66	ABCC11	ATP-binding cassette sub-family C member 11
P35580	MYH10	Myosin-10
P53355	DAPK1	Death-associated protein kinase 1
O76039	CDKL5	Cyclin-dependent kinase-like 5
P62826	RAN	GTP-binding nuclear protein Ran
P09529	INHBB	Inhibin beta B chain
P67809	YBX1	Nuclease-sensitive element-binding protein 1
P07355	ANXA2	Annexin A2
Q96BF6	NACC2	Nucleus accumbens-associated protein 2
Q86YH2	ZNF280B	Zinc finger protein 280B
Q9NW97	TMEM51	Transmembrane protein 51
Q9UGU0	TCF20	Transcription factor 20
Q9Y2S6	TMA7	Translation machinery-associated protein 7
O60861	GAS7	Growth arrest-specific protein 7

### Functional analysis of all PA interacting proteins

**Figure 2:**
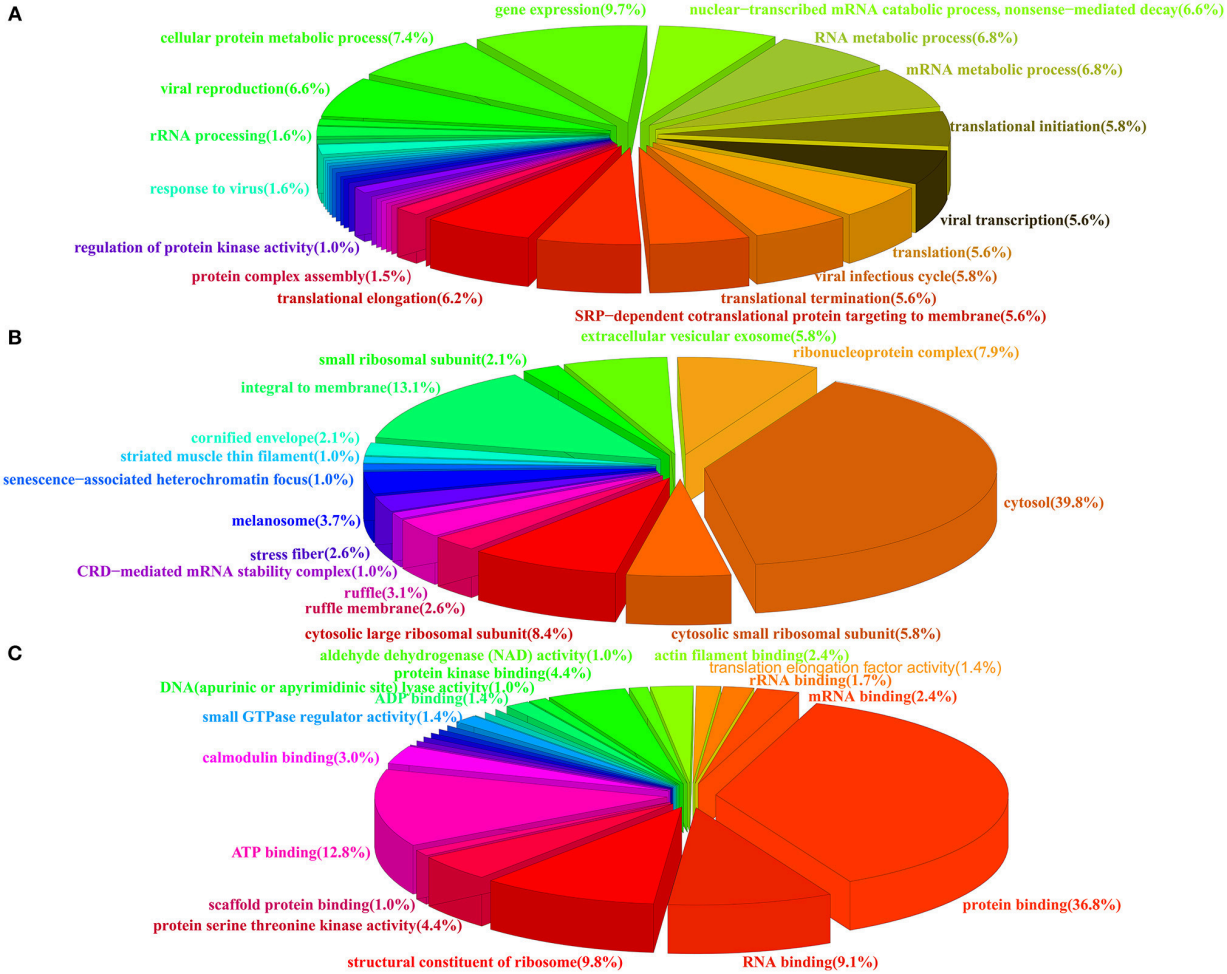
**Pie charts showing the GO annotation of the identified cellular proteins**. The GO annotation was analyzed by DAVID database and the percentage of each GO component was shown. **(A)** Biological process. **(B)** Cellular components. **(C)** Molecular function.

To obtain an overall functional profile of the interactome associated with the PA protein, the 278 identified proteins were assigned for further bioinformatics analysis. As shown in Figure 2, three major types of annotations, biological process, cellular components, and molecular functions, were generated in the GO consortium website. In addition, we found that subclasses associated with gene expression, viral infectious cycle and viral transcription were highly enriched in biological process category (Figure 2A). As for the most enriched subclasses in cellular component, the major types, such as cytosol, integral to membrane, and ribonucleoprotein complex, were enriched (Figure 2B). Moreover, molecular function analysis showed that protein binding, ATP binding, structural constituent of ribosome and RNA binding were highly enriched (Figure 2C). A more detailed summary of GO annotation of the identified proteins was provided in Table S2.

**Figure 3:**
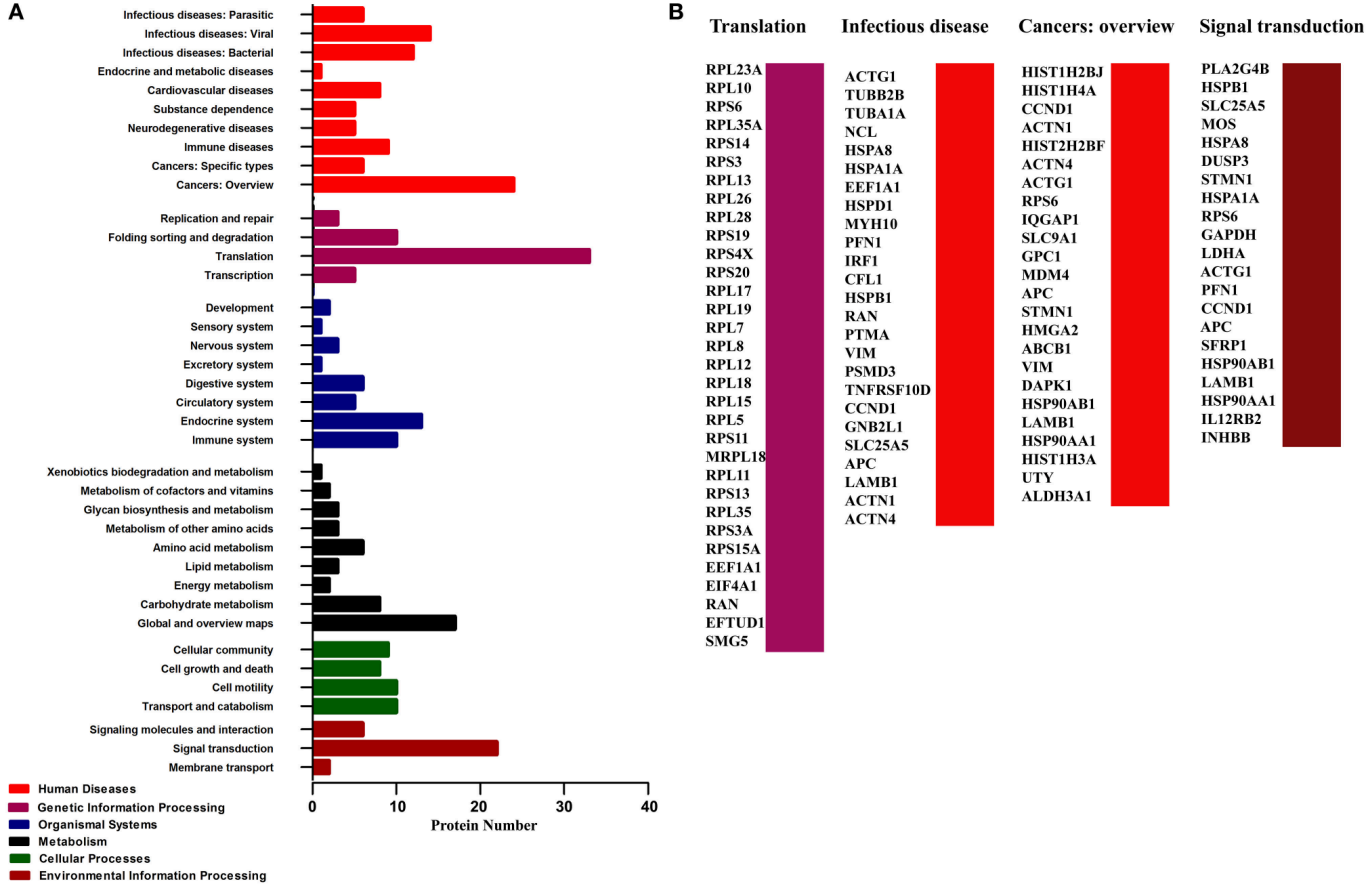
**Pathway analysis of the cellular proteins interacting with PA based on KEGG. (A)** Classification of the enriched KEGG pathways of the identified proteins. **(B)** Name of the identified proteins related with the top four KEGG pathways classification.

In addition, KEGG pathway analysis revealed an enrichment of 165 pathways related with 130 proteins of the infection network (Table S3). Six types of pathways were covered, including Human Diseases, Genetic Information Processing, Organismal Systems, Metabolism, Cellular Processes, and Environmental Information Processing (Figure 3A). To be noted, pathways involved in translation, infectious disease (contains infecious disease: Viral, infecious disease: Bacterial and infecious disease: Parasitic), cancers: overview and signal transduction were highly enriched in the PA-host interactome (Figure 3B, Table S3).

### Detalied analysis of the two targeted PA-host interacting proteins

**Figure 4:**
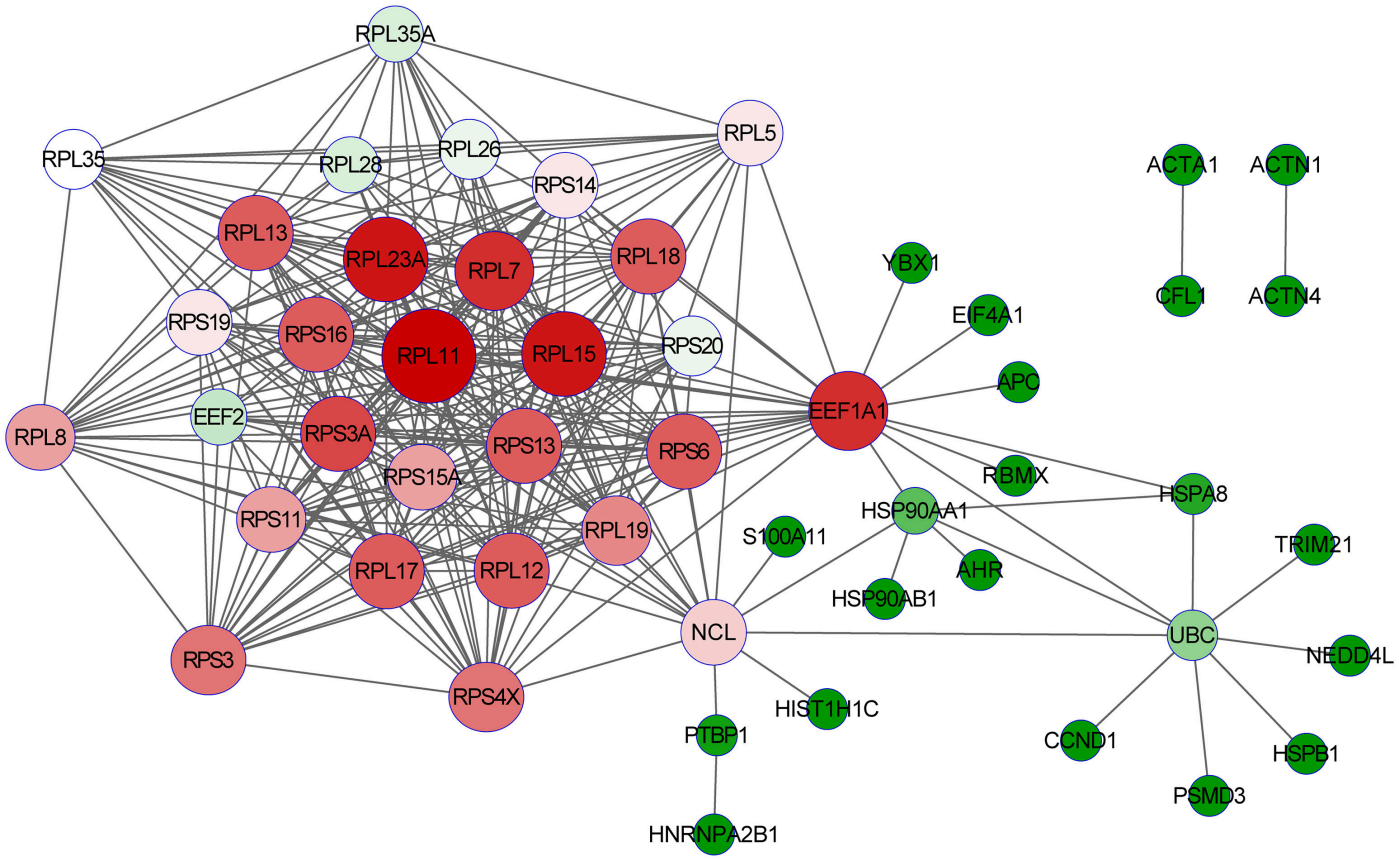
**The interaction network of the identified proteins with NCL and eEF1A1**. The open source Bioinformatics software Cytoscape 3.2.0 (http://www.cytoscape.org/) was used to visualize protein-protein interactions.

After detatied functional analysis, we then focused on two targeted novel cellular partners (NCL and eEF1A1) that might interact with PA, cellular proteins. As shown in Figure 4, the interactome profile indicated the important protein-protein interactions of the identified proteins associated with NCL and eEF1A1. The analysis revealed that NCL and eEF1A1 are cross-linked with several proteins with interesting interactions, including HNRNPA2B1-PTBP1-NCL-RPL5 and AHR-HSP90AA1-EEF1A1-RPL5. By GO annotation, we identified that these proteins have important functions in RNA transport, RNA splicing, apoptotic process, and translation which are closely asociated with IAV infection.

**Figure 5:**
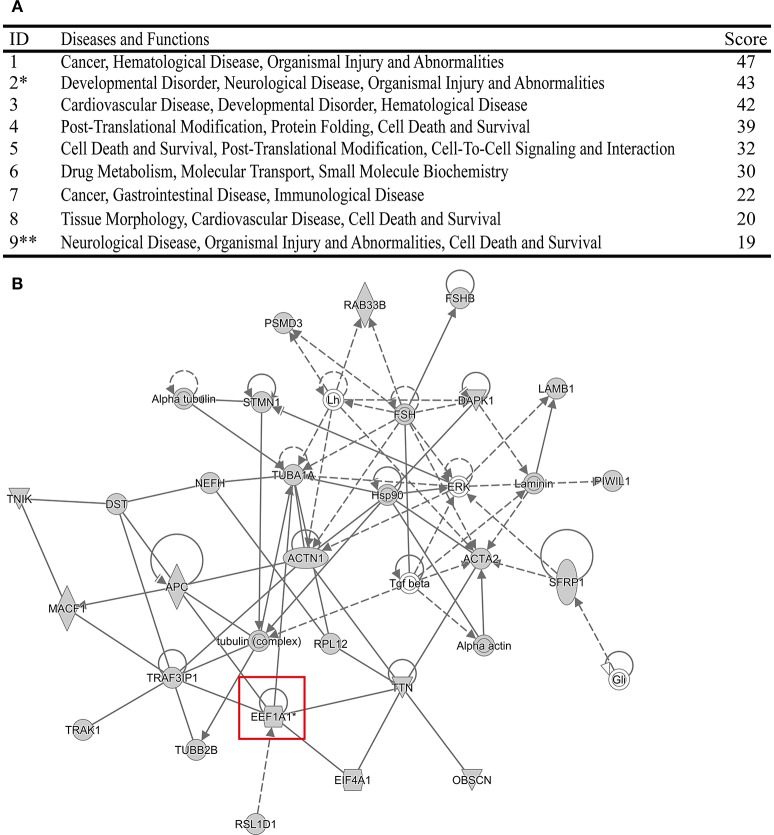
**The top Diseases and Functions of the identified cellular proteins analyzed using IPA program. (A)** ^*^Stands for that the Diseases and Functions related with eEF1A1. ^**^Stands for that the Diseases and Functions related with NCL. **(B)** Network of the Diseases and Functions related with eEF1A1.

Meanwhile, the top Diseases and Functions analysis of the identified 278 cellular proteins was also performed using IPA software (Figures 5A,B, Figure S1). Surprisingly, we found that both NCL and eEF1A1 were related with Organismal Injury and Abnormalities. In addition, NCL was also involved in Cell Death and Survival. It has been demonstrated that immune injury and cell death contribute to the pathogenicity of IAV (Iwai et al., 2013; Duan and Thomas, 2016; Sridhar, 2016). Therefore, these resluts indicated that NCL and eEF1A1 might contribute to the virulence of IAV.

### Comparison of the identifed PA-host interacting proteins with the available interactome data

**Figure 6:**
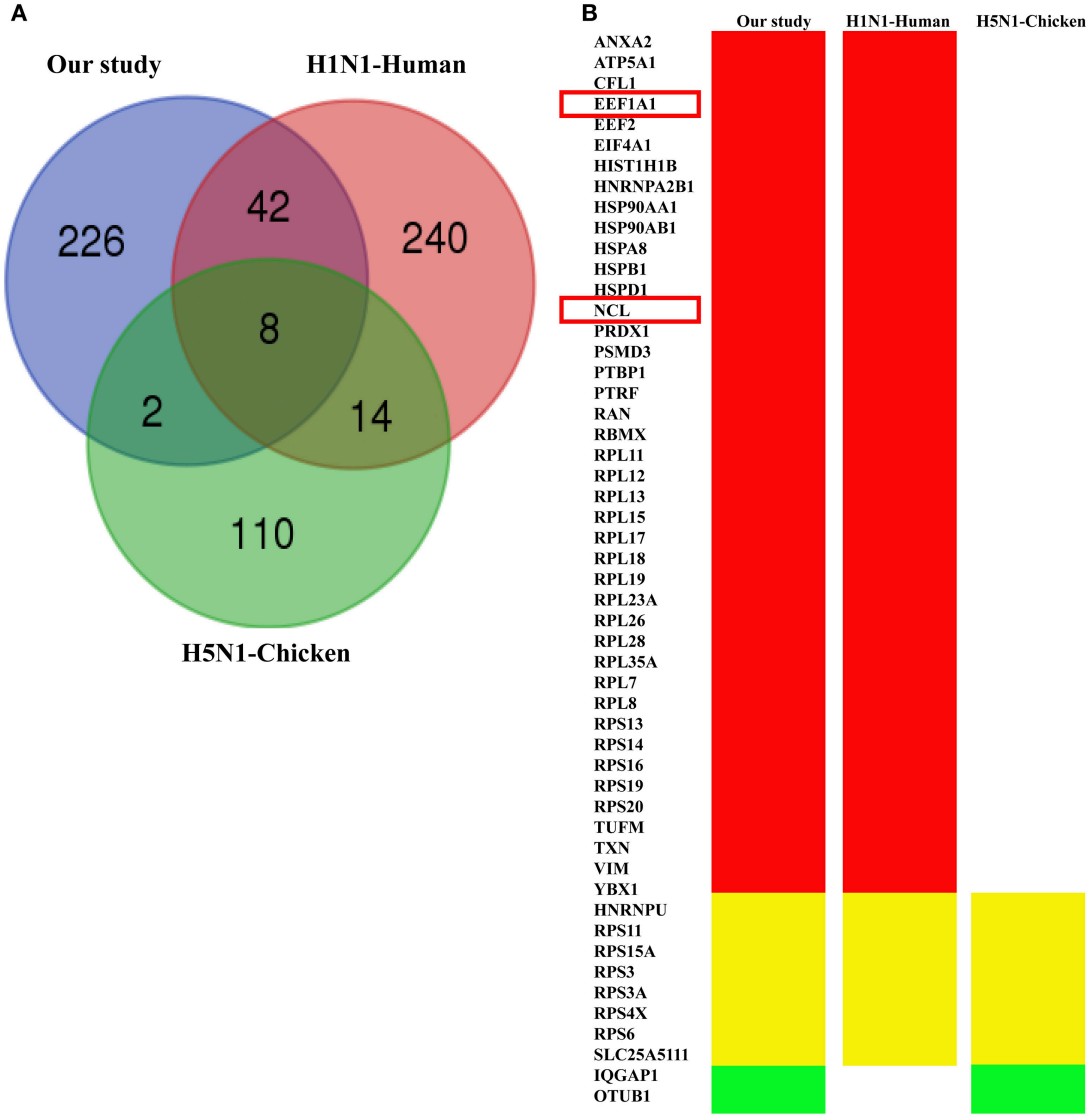
**Shared host interacting factors of the PA protein across different hosts and IAV strains. (A)** Venn diagram showing shared host interacting proteins with PA among our study, PA associated proteins of H1N1 IAV in the A549 cell line, and of H5N1 IAV in the DF1 cell line. **(B)** Name of the proteins shared by three different studies. Red stands for that the proteins only shared by our study and H1N1-Human study. Yellow represents the proteins shared by these three studies. Green stands for the proteins only shared by our study and H5N1-Chicken study. The NCL and eEF1A1 (highlighted in the red box) were found both in our study and H1N1-Human study.

In order to systematically analyze the screened PA-interacting host proteins, we then made a detailed comparison of the interactome data associated with PA from different studies. Using affinity purification-mass spectrometry (AP-MS), Wang et al. identified 134 H5N1 IAV PA interacting proteins in chicken cells (Wang et al., 2016). Watanabe et al. also characterized 304 proteins that interact with the PA protein of the H1N1 influenza virus in human cells using LC/MS (Watanabe et al., 2014). In the present study, 278 human proteins were found to interact with H5N1 PA protein using LC-MS/MS in A549 cells. As a result, our dataset shared 50 and 10 host proteins with data from H1N1-Human study and H5N1-Chicken study respectively (Figure 6A; Table S4). It is worth noting that NCL and eEF1A1 are identifed as PA-interacting factors both in our study and H1N1 study (Figure 6B). These resluts further indicated that these two proteins may play an important role in influenza virus infection.

### Validation of interaction between NCL, eEF1A1, and PA using co-IP and co-localization

**Figure 7:**
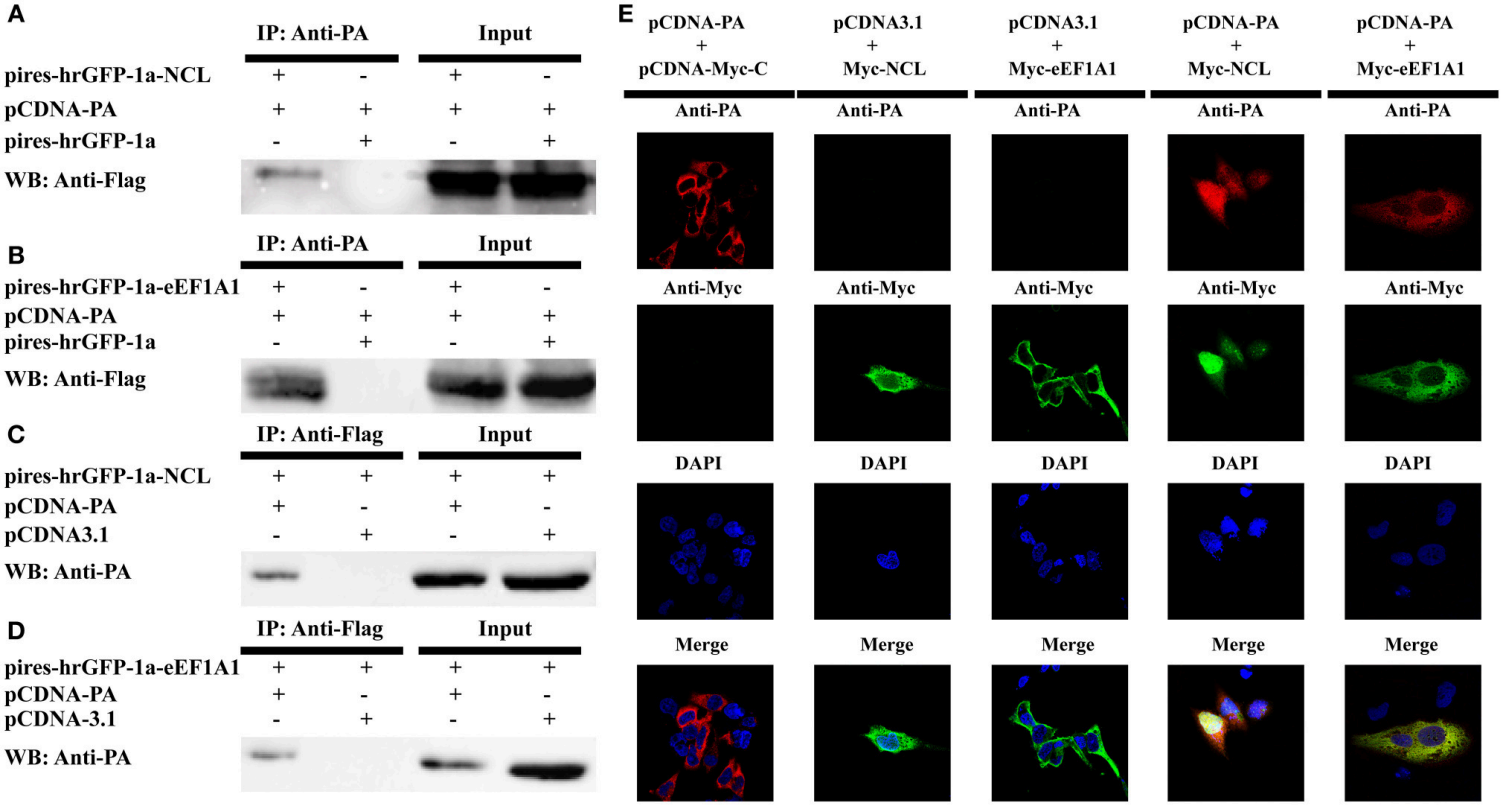
**Confirmation of the interaction of IAV PA with NCL and eEF1A1**. 293T cells were transiently co-transfected with the PA-expressing plasmid or the empty vector and the tagged NCL, eEF1A1 or the empty vector for co-immunoprecipitation and co-localization studies. **(A–D)** Cell lysates were prepared at 48 h post-transfection and the proteins were immunoprecipitated with anti-PA or anti-FLAG antibodies. Proteins in cell lysates (input) and immunoprecipitated samples were detected with the antibodies against FLAG or PA in Western blot. **(A)** Co-precipitation of about 110 kDa NCL protein with recombinant viral PA in cell lysate. **(B)** Co-precipitation of about 50 kDa eEF1A1 protein with PA in cell lysate. **(C)** Co-precipitation (reverse IP) of about 85 kDa PA with NCL. **(D)** Co-precipitation (reverse IP) of about 85 kDa PA with eEF1A1. **(E)** Confocal microscopy analysis was carried out for demonstrating colocalization of PA and NCL or eEF1A1. 293T cells were transiently co-transfected with PA expressing vector or empty vector and Myc-tagged NCL expressing vector, eEF1A1 expressing vector or empty vector, respectively. 24 h later, cells were fixed, and yellow regions are the areas of PA and NCL or eEF1A1 co-localization. Nuclei were stained using DAPI (blue).

Considering the NCL and eEF1A1 were shared in this study and the H1N1 study, we then verifed their interactions with the PA protein. 293T cells were co-transfected with pCDNA-PA or the empty vector (pCDNA3.1) alone with the Flag-tagged NCL expression plasmid (pires-hrGFP-1a-NCL), Flag-tagged eEF1A1 expression plasmid (pires-hrGFP-1a-eEF1A1) or the empty vector (pires-hrGFP-1a) respectively. After 48 h post-transfection, the co-IP assay was performed using anti-PA or anti-FLAG antibody. The immune-complexes were detected in western blot assay using anti-FLAG or anti-PA antibody subsequently. The results showed that both NCL and eEF1A1 were detected only in the presence of PA, but not in the presence of empty vector (Figures 7A,B). In addition, the PA protein was detected only in the presence of NCL and eEF1A1, but not in the presence of empty vector (Figures 7C,D).

To demonstrate the co-localization of NCL and PA or eEF1A1 and PA, three plasmid-pCDNA-Myc-NCL, pCDNA-Myc-eEF1A1, and pCDNA-PA were constructed. Cells grown on coverslips were co-transfected with pCDNA-PA or the empty vector (pCDNA3.1) alone with pCDNA-Myc-NCL, pCDNA-Myc-eEF1A1, or the empty vector (pCDNA-Myc-C), respectively. The cells were fixed at 24 h post-transfection. Then the cells were subjected to confocal microscopy analysis by using primary antibodies of Myc-tag, PA and secondary antibodies with two different fluorophores. Co-localization of two proteins in cells was visualized by confocal microscope. As shown in Figure 7E, NCL and PA were co-localized both in the nucleus and cytoplasm, while eEF1A1 and PA were only detected in the cytoplasm of the cell. Thus, these results clearly confirmed that the PA protein interacts well with the cellular proteins NCL or eEF1A1.

## Discussion

Since the first report in Asia in 1997, H5N1 influenza viruses have spread to many countries on different continents and caused considerable loss to both poultry industry and human health (Webster and Govorkova, 2006). In addition, IAV can evade the host immune response and cause persistent infection (Quinones-Parra et al., 2014). However, until now, the mechanism of IAV infection is not fully elucidated. It is well-known that virus can infect hosts and survive in host cells through interacting with the host cellular factors, exploiting the cellular pathways, and subverting defense systems inhibiting viral propagation established by host cells (Lamkanfi and Dixit, 2010; Watanabe et al., 2010). PA, a subunit of RNA polymerase of IAV, possesses pleiotropic effects: (1) plays important roles in viral transcription and replication (Kawaguchi et al., 2005; Regan et al., 2006). (2) contributes to the adaption of IAV in mammalian host (Gabriel et al., 2005; Sakabe et al., 2011; Seyer et al., 2012). (3) increases the virulence of IAV (Hulse-Post et al., 2007; Song et al., 2011; Hu et al., 2013a). (4) involves in host protein shut off (Rodriguez et al., 2007; Desmet et al., 2013; Llompart et al., 2014). (5) participates in immune regulation in the process of IAV infection (Huang et al., 2013; Sakabe et al., 2013). Furthermore, three novel ORFs derived from the PA gene encoding PA-X (Jagger et al., 2012), PA-N155, and PA-N182 (Muramoto et al., 2013) have been identified. Especially, PA-X plays important roles in viral life cycle and pathogenesis (Hu et al., 2015). Therefore, illustrating the PA-host interactions that might contribute to viral pathogenesis of IAV is crucial for understanding the potential mechanisms of IAV infection and development of new anti-viral drugs.

Many technologies have been employed to characterize the protein interaction network, such as AP-MS and yeast two-hybrid (Y2H). Currently, compared with Y2H, AP-MS is more widely-used because it could reveal interactions that mimic the actual physiological condition. In this study, IP coupled LC-MS/MS technique was applied to explore the host factors in IAV-infected A549 cells that interact with PA of the H5N1 influenza virus. Compared with IP assay using PA plasmid-transfected cells, interactome data obtained from virus-infected cells could provide insights for this method not only conserved the native protein conformation during virus replication, but also able to explore the cellular proteins that might interact indirectly with PA protein.

In this study, 278 human cellular proteins were identified to interact with PA of H5N1 influenza virus (Table 1, Table S1). To further explore the biological significance of the interaction between PA and host cellular proteins, bioinformatics analysis was utilized to comprehensively evaluate and characterize the bio-functions of the identified host proteins. Results of sub-cellular locations and functional classes analysis based on GO analysis demonstrated that the identified proteins were highly associated with gene expression, viral translation, and replication (Figure 2).

KEGG pathway analysis showed that 130 of 278 identified host proteins involved in 165 pathways. Notably, a significant proportion of the enriched KEGG pathways, including 4 enriched KEGG pathways and 33 proteins, were shown to be associated with translation (Figure 3B, Table S3). Therefore, we surmised that the translation-related pathways enriched by PA-host interactions might play an important role in IAV infection. Regarding IAV life cycle, after entry into the host cells, the viral ribonucleoprotein complexes and viral RNA (vRNPs) are transported into the nucleus where replication occurs. In the nucleus, the polymerase also allows the transcription of the genome into mRNA, which is then transported back to the cytoplasm and translated into viral proteins. Then, the NP, PB1, PB2, and PA proteins will re-enter the nucleus to form the RNP complex with viral RNA (Berri et al., 2013). Therefore, our functional analysis data of the PA-host interaction proteins further confirmed the important role of PA in viral replication. The second highest proportion of enriched KEGG pathways was associated with infectious disease, including 16 enriched KEGG pathways and 25 proteins (Figure 3B, Table S3). Among the 25 proteins, ACTG1, HSPA8, TNFRSF10D, and HSPA1A are highly related with the pathway of IAV (Table S3). Therefore, we speculated that the remaining 21 proteins associated with infectious disease may play a shared role in influenza virus infection and other infectious pathogens. Thus, we surmised that studies in terms of other etiologies may offer good ways to investigate the pathogenesis of IAV.

Among the 278 identified host proteins, NCL and eEF1A1 were confirmed to interact with PA using co-IP and co-localization (Figure 7). NCL is a multi-functional protein that predominantly locates in the cell nucleolus. Although NCL is also found on the cell surface, it does not possess a transmembrane domain and therefore it may not act as a typical membrane protein (Hovanessian et al., 2000). Previous studies have demonstrated that NS1, HA, and NP proteins of IAV can also interact with NCL. The NS1 protein interacts with NCL in nucleoplasm and nucleolus mainly via its C-terminal NLS2/NoLS and N-terminal NLS1 domains (Melén et al., 2012). Chan et al. revealed that HA protein is also associated with NCL (Chan et al., 2016). Both inhibiting cell surface NCL and depleting endogenous NCL can substantially reduce influenza virus internalization. In addition, Kumar et al. identified the interaction between NCL and NP. Down-regulation of the host NCL expression facilitates the viral gene transcription, and the viral replication (Kumar et al., 2016). Moreover, it has been demonstrated that NCL also plays a significant role in the internalization of parainfluenza virus type 3 (Bose et al., 2004). Furthermore, surface NCL is also shown to be involved in the entry of several viruses, including respiratory syncytial virus (RSV), CrimeanCongo hemorrhagic fever virus (CCHFV), and human immunodeficiency virus (HIV) (Nisole et al., 2002; Tayyari et al., 2011; Xiao et al., 2011). However, the potential roles of the NCL in the PA-associated functions are currently unknown and need to be further confirmed in the future.

eEF1A1 is not only a translation factor but also a pleiotropic protein, including cytoskeleton modulation, chaperone-like activity, and regulation of cell proliferation and cell death (Abbas et al., 2015). Previous studies have indicated that eEF1A1 is a p53-interacting protein and has anti-apoptotic property in p53 family signaling pathway (Blanch et al., 2013). P53 also interplays with influenza virus, and down-regulation of p53 expression results in resisting host innate and adaptive immune system against IAV (Muñoz-Fontela et al., 2011; Terrier et al., 2011, 2012). However, the mechanism underlying this activity is still poorly understood. Therefore, we surmised that the p53 interacting protein eEF1A1 identified in this study might act as a p53 partner and facilitate the IAV infection. However, further studies are needed to verify this assumption.

In summary, in this study, 278 cellular proteins were identified as interacting partners for PA of H5N1 IAV. GO and KEGG pathway analysis revealed that these proteins played important roles in translation and viral replication. Further IPA analysis demonstrated that these host factors are associated with Organismal Injury and Abnormalities, Post-Translational Modification, Protein Folding, Cell Death, and Survival. Moreover, two targeted host proteins, NCL and eEF1A1, associated with Organismal Injury and Abnormalities and Cell Death and Survival, were further confirmed to interact with PA both using co-IP and co-localization analysis. Therefore, our findings provide new targets for further research of IAV infection and pathogenesis.

## Author contributions

Conceived and designed the experiments: ZG, JH and XFL. Performed the experiments: ZG, YYL, QY, KY and JH. Analyzed the data: ZG and JH. Contributed reagents/materials/analysis tools: DL, XQW, MG, ZLH, SLH, SJC, XWL, HML, DXP, XAJ and WBL. Wrote the paper: ZG, JH and XFL.

## Funding

This work was supported by the National Natural Science Foundation of China (31502076), by the Jiangsu Provincial Natural Science Foundation of China (BK20150444), by the Natural Science Foundation of the Higher Education Institutions of Jiangsu Province, China (15KJB230006), by the Special Financial Grant from the China Postdoctoral Science Foundation (2016T90515), by the National key research and development project of China (2016YFD0501601 and 2016YFD0500202), by the National Key Technologies R&D Program of China (2015BAD12B01-3), by the earmarked fund for Modern Agro-industry Technology Research System (nycytx-41-G07) and by A Project Funded by the Priority Academic Program Development of Jiangsu Higher Education Institutions, (PAPD).

### Conflict of interest statement

The authors declare that the research was conducted in the absence of any commercial or financial relationships that could be construed as a potential conflict of interest.
