# Supplementary figures and images for: Generation and Comprehensive Analysis of Host Cell Interactome of the PA Protein of the Highly Pathogenic H5N1 Avian Influenza Virus in Mammalian Cells
Source: Front Microbiol. 2017 Apr 28;8:739. doi: 10.3389/fmicb.2017.00739 (PMC5408021; doi:10.3389/fmicb.2017.00739)

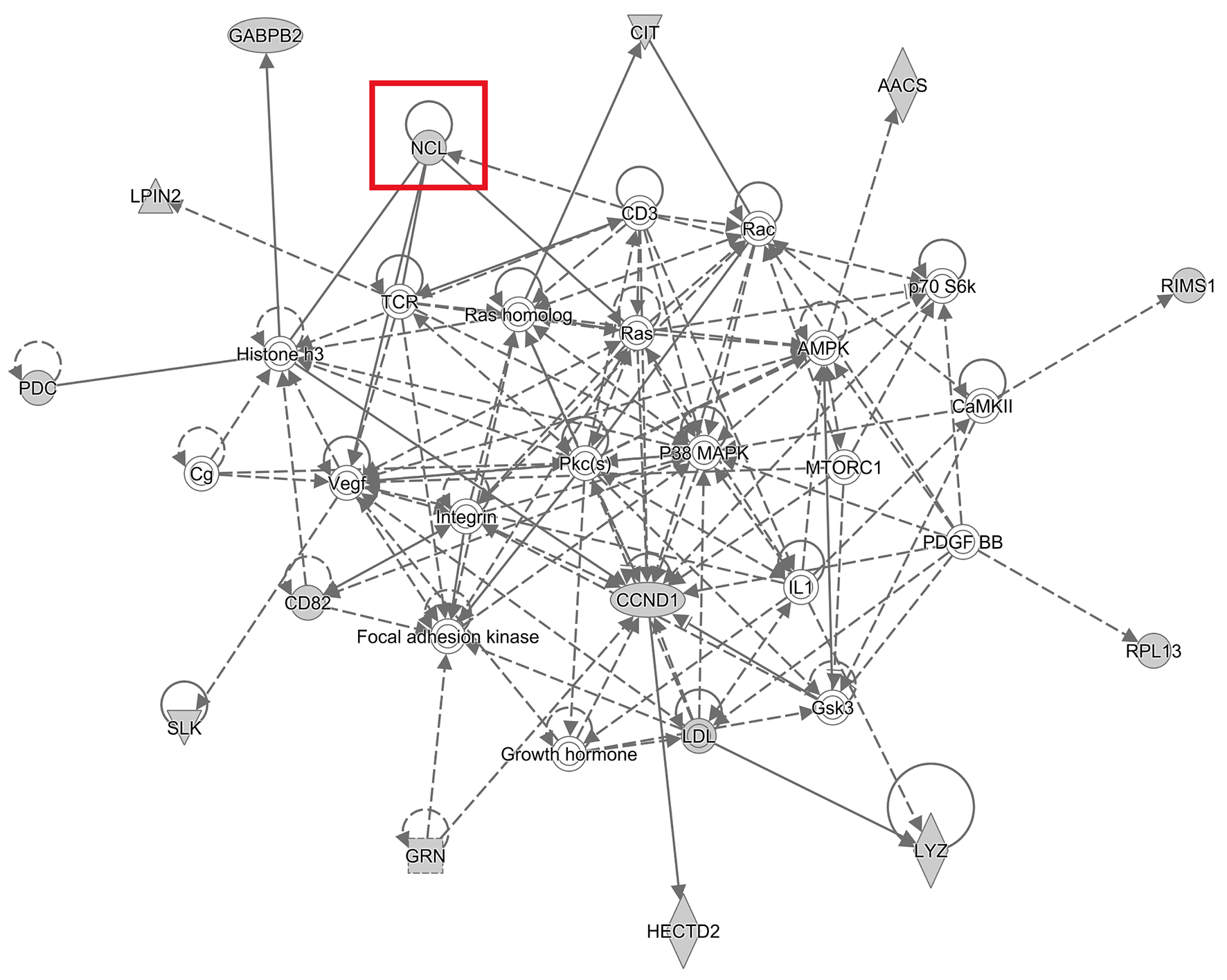

Supplement: Supplementary file 5 [file Image1.TIF]
